# Opioid prescription patterns among radiation oncologists in the United States

**DOI:** 10.1002/cam4.2907

**Published:** 2020-03-13

**Authors:** Tina Q. Huang, Eric M. Chang, Tristan R. Grogan, Emily J. Martin, Ann C. Raldow

**Affiliations:** ^1^ David Geffen School of Medicine University of California Los Angeles CA USA; ^2^ Department of Radiation Oncology University of California Los Angeles CA USA; ^3^ Department of Medicine University of California Los Angeles CA USA

**Keywords:** cancer pain, drug prescriptions, opioid consumption, opioids, prescription drug overuse, radiation oncology

## Abstract

**Background:**

Radiation oncologists (ROs) play an important role in managing cancer pain; however, their opioid prescribing patterns remain poorly described.

**Methods:**

The 2016 Medicare Physician Compare National Downloadable and the 2016 Medicare Part D Prescriber Data files were cross‐linked to identify RO‐written opioid prescriptions.

**Results:**

Of 4,627 identified ROs, 1,360 (29.3%) wrote >10 opioid prescriptions. The average number of opioid prescriptions written was significantly (*P* ≤ .05) associated with the following RO characteristics: sex [13.1 ± 36.5 male vs 7.5 ± 16.9 female]; years since medical school graduation [4.5 ± 11.5 1‐10 years vs 12.6 ± 26.0 11‐24 years vs 13.3 ± 40.9 ≥25 years]; practice size [15.5 ± 44.6 size ≤10 vs 13.3 ± 25.9 size 11‐49 vs 8.5 ± 12.7 size 50‐99 vs 8.8 ± 26.9 size ≥100]; Medicare Physician Quality Reporting System (PQRS) participation [12.6 ± 31.8 yes vs 7.0 ± 35.4 no]; and practice location [17.4 ± 47.0 South vs 10.6 ± 29.4 Midwest vs 8.1 ± 13.9 West vs 6.9 ± 15.2 Northeast]. On multivariable regression modeling, male sex (RR 1.29, 95% CI 1.22‐1.35, *P* < .001), ≥25 years since graduation (RR 0.78, 95% CI 0.64‐0.70, 1‐10 years vs ≥25 years; RR 1.00, 95% CI 0.96 ‐ 1.04, 11‐24 years vs ≥25 years; *P* < .001), practice size <10 members (RR 1.51, CI 1.44‐1.59, ≤10 vs ≥100 members, RR 1.27, CI 1.20‐1.34, 10‐49 vs ≥100 members, RR 0.86, CI 0.80‐0.92, 50‐99 vs ≥100 members, *P* < .001), PQRS participation (RR 1.12, CI 1.04‐1.19, *P* < .002), and Southern location (RR 0.67, CI 0.64‐0.70, Midwest vs South; RR 0.39, CI 0.37‐0.41, Northeast vs South; RR 0.43, CI 0.41‐0.46, West vs South; *P* < .001) were predictive of higher opioid prescription rates.

**Conclusions:**

Factors associated with increased number of RO‐written opioid prescriptions were male sex, ≥25 years since graduation, group practice <10, PQRS participation, and Southern location. Additional research is required to establish optimal opioid prescribing practices for ROs.

## Graphical abstract

The number of opioid prescriptions written by radiation oncologists are related to specific physician and practice characteristics; further investigation is needed to establish opioid prescribing best practices within radiation oncology.

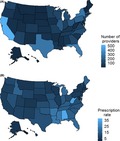

## 1 INTRODUCTION

In 2017, the Department of Health and Human Services declared the opioid crisis in the United States (US) a national emergency. Since then, several government agencies have released new guidelines aimed at reducing opioid prescriptions.^1^, ^2^ Medicare drug benefit plans now require beneficiaries to only use selected prescribers or pharmacies for opioid prescriptions, and state‐specific laws now require physicians to consult the Controlled Substance Utilization Review and Evaluation System (CURES) before prescribing Schedule II‐IV controlled substances.

While cancer patients are meant to be exempt from these policies, efforts to curb opioid usage for chronic, nonmalignant pain have had unintended consequences on cancer patients requiring opioids for adequate symptom relief. The stigma of opioid use and fears of opioid addiction are pervasive, influencing patients, families, and physicians. At the same time, cancer pain remains chronically undertreated, with a third of patients not receiving adequate analgesia.^3^

Radiation oncologists (ROs) play an important role in managing cancer‐related pain; however, their opioid prescribing patterns are not well described. Previous studies examining ROs’ attitudes toward pain management found that while the majority of ROs recognized that most cancer patients with long‐term pain are undertreated, 40% rated management of cancer‐related pain in their practice as fair to poor.^4^, ^5^ Furthermore, despite efforts to promote pain management education among ROs, comfort with pain assessment and management has not improved.^5^ The aim of this study was to characterize the prescribing behaviors of US ROs and to assess whether these behaviors are associated with certain physician and practice characteristics.

## 2 METHODS

This study used federally designated public use files and no private identifiable information was obtained. This study was exempt from review by the University of California, Los Angeles Institutional Review Board.

### 2.1 Physician cohort identification

The most recent Medicare Physician Compare National Downloadable File (2016) contains general demographic information on all Medicare‐accepting practicing physicians in the US We identified ROs in this file using their designated primary specialty. For each physician, National Provider Identifier (NPI), sex, year of medical school graduation, group practice identifier, and zip code were extracted.

### 2.2 Prescription identification

We used the 2016 Medicare Provider Utilization and Payment Data: Part D Prescriber Public Use File database from the Centers for Medicare and Medicare Services (CMS) for prescription identification. This database identifies physicians by their NPIs and contains information on prescription drugs prescribed to Medicare Part D beneficiaries, including brand name, generic name, and total number of prescriptions (original prescriptions and refills). The Medicare Part D prescription plan covers approximately 70% of all Medicare beneficiaries.^6^ In order to maintain patient privacy, CMS excludes drugs and providers with 10 or fewer total attributable claims over the course of the year. Similarly, beneficiary counts, claim counts, 30‐day fill counts, drug costs, and days’ supply were suppressed if the value was 10 or less.

### 2.3 Physician characteristics and prescription data set cross‐linking

Using NPIs for all identified ROs, data from the Part D Prescriber Public Use File (PUF) were cross‐linked with the 2016 Medicare Physician Compare National Downloadable File, thereby permitting identification of RO‐written Medicare prescriptions. For ROs who prescribed more than 10 opioid prescriptions, we extracted the following information: total prescriptions written (original and refills); total opioid prescriptions written (original and refills); total days’ supply of opioids; and opioid prescribing rate (the percentage of all prescriptions that were for opioids). Average days’ supply of opioid prescriptions was also computed. For physicians who wrote 10 or fewer opioid prescriptions in 2016, we used a value of 5 for number opioid prescriptions per physician, following procedures recommended by CMS and implemented by previous investigators.^1^, ^7^, ^8^ ROs not identified in the Part D PUF were assigned a value of 0 opioid prescriptions. For ROs identified in the 2016 Medicare Physician Compare National Downloadable File, we extracted the following physician information: sex; year of medical school graduation; group practice size (total number of individual professionals affiliated with a group, including non‐ROs); participation in the Medicare Physician Quality Reporting System (PQRS); and state (classified into geographic regions matching designations from the US Census Bureau).^9^

### 2.4 Statistical analysis

Characteristics of opioid‐prescribing ROs were reported using means (Standard Deviation, SD) for continuous measures and frequency (%) for categorical variables. Opioid prescription comparisons between groups were made using the t‐test for variables with two levels or the one‐way ANOVA for variables with three or more levels (eg, region). Most commonly prescribed opioids were identified with available drug‐specific information. Prescribing behavior between the various ROs and practice characteristics was summarized and formally tested using the *t* test. Multivariable linear regression was performed to identify factors independently associated with the number of opioid prescriptions. As a sensitivity analysis, we ran a separate multivariable negative binomial model using the same predictors for the number of opioid scripts prescribed by each RO (with an offset to account for total number of scripts). Results from this model were presented as incidence rate ratios (Table 3). Statistical analyses were performed using SPSS V25 (IBM Corp.). A heatmap of ROs oncologists in the US and opioid prescription rates for the US was constructed using the “usmap” package in R V3.5.1.

## 3 RESULTS

The characteristics of all identified ROs are shown in Table 1. Of 4627 ROs, 3,405 (73.6%) were male and 1222 (26.4%) were female. ROs were approximately evenly distributed through the US, with the highest number of ROs in the South (n = 1,574, 34.2%), followed in number by the Midwest (n = 1066, 23.1%), West (n = 1032, 22.4%), and Northeast (n = 934, 20.3%). The majority of ROs practice in groups with more than 100 members (n = 2361, 52.9%), followed in number by ≤ 10 members (n = 985, 22.1%), 11‐49 members (n = 742, 16.6%), and 50‐99 members (n = 371, 8.3%). The median practice size among all identified ROs was 138 members.

**Table 1:** Participant characteristics (n = 4627) and opioid prescription behavior, stratified by physician characteristics

Characteristics	Category	No., (%)	Mean opioids prescribed (SD)	*P*‐value
Sex	Female	1222 (26.4)	7.53 (16.9)	<.001
	Male	3405 (73.6)	13.06 (36.5)	
Years since medical school	1‐10	764 (16.6)	4.5 (11.5)	<.001
	11‐24	1672 (36.3)	12.6 (26.0)	
	> 25	2168 (47.1)	13.3 (40.9)	
Number members in group	<10	985 (22.1)	15.5 (44.6)	<.001
	10‐49	742 (16.6)	13.3 (25.9)	
	50‐99	371 (8.3)	8.5 (12.7)	
	>100	2361 (52.9)	8.8 (26.9)	
Quality measures	Reports	3785 (82.8)	12.6 (31.8)	<.001
	Does not report	842 (18.2)	6.96 (35.4)	
Region	Midwest	1066 (23.1)	10.6 (29.4)	<.001
	Northeast	934 (20.3)	6.9 (15.2)	
	South	1574 (34.2)	17.4 (47.0)	
	West	1032 (22.4)	8.1 (13.9)	

Abbreviation: SD, standard deviation.

**Figure 1:**
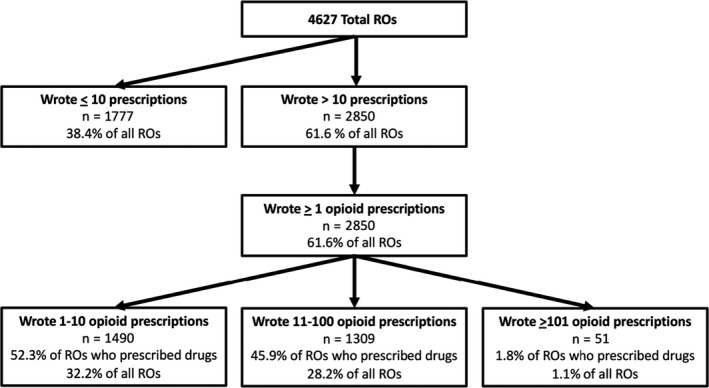
Flowchart of Medicare‐accepting RO drug and opioid prescription behavior. RO, radiation oncologist

In total, 2,850 (61.6%) ROs wrote at least one opioid prescription (Figure 1). Of these ROs, 1,490 (52.3%) wrote 1‐10 opioid prescriptions, 1,309 (45.9%) wrote 11‐100 opioid prescriptions, and 51 (1.8%) wrote greater than 100 opioid prescriptions. Overall, ROs wrote an average of 11.6 SD(±)32.6 opioid prescriptions. Among ROs who prescribed an opioid at least once, the average number of opioid prescriptions written increased to 18.8 ± 39.8. ROs who prescribed more than 10 opioids prescribed an average of 34.0 ± 53.7 opioids), with an average day supply of 10.5 ± 5.4 days. The average opioid prescribing rate (percent of total prescriptions written that were opioid prescriptions) among ROs who had written more than 10 total prescriptions was 19.2% ±27.7%. For ROs who wrote more than 10 opioid prescriptions, the average opioid prescription rate was 40.1% ± 27.7%. Table 2 identifies all opioids identified in the database and the number of prescriptions for each opioid. The four most commonly prescribed opioids were: hydrocodone with acetaminophen (n = 23 577; oxycodone (n = 10 226); oxycodone with acetaminophen (n = 5,310); and fentanyl (n = 4436); and morphine (n = 2162).

**Table 2:** Summary table of opioids prescribed by radiation oncologists

Opioid	Total claims
Hydrocodone/Acetaminophen	23 577
Oxycodone HCl	10 226
Oxycodone HCl/Acetaminophen	5310
Fentanyl	4436
Morphine Sulfate	2162
Tramadol HCl	951
Acetaminophen With Codeine	816
Hydromorphone HCl	445
Methadone HCl	171
Tramadol HCl/Acetaminophen	33
Buprenorphine HCl	26
Hydrocodone/Ibuprofen	25
Oxymorphone HCl	24
Tapentadol HCl	15
Butalbit/Acetamin/Caff/Codeine	12
Meperidine HCl	12
Opium Tincture	12

The average number of opioid prescriptions per RO was significantly (*P* < .001) associated with the following physician and practice characteristics: sex [13.1 ± 36.5 male vs 7.5 ± 16.9 female]; years since medical school graduation [4.5 ± 11.5 1‐10 years vs 12.6 ± 26.0 11‐24 years vs 13.3 ± 40.9 ≥25 years]; group practice size [15.5 ± 44.6 size <10 vs 13.3 ± 25.9 size 11‐49 vs 8.5 ± 12.7 size 50‐99 vs 8.8 ± 26.9 size ≥100]; PQRS participation [12.6 ± 31.8 yes vs 7.0 ± 35.4 no]; and practice location [17.4 ± 47.0 South vs 10.6 ± 29.4 Midwest vs 8.1 ± 13.9 West vs 6.9 ± 15.2 Northeast] (Table 1).

Two multivariable regression models (negative binomial and linear) for the rate of opioid prescriptions were constructed (Table 3 and Table S1) and gave similar results. Multivariable negative binomial modeling showed a significant independent association with the average number of opioid prescriptions written per RO and male sex compared to female providers (RR 1.29, 95% CI 1.22‐1.35, *P* < .001), at least 25 years since medical school graduation (RR 0.78, 95% CI 0.64‐0.70, 1‐10 years vs ≥25 years; RR 1.00, 95% CI 0.96‐1.04, 11‐24 years vs ≥25 years; *P* < .001), group practice size ≤ 10 members (RR 1.51, CI 1.44‐1.59, ≤10 vs ≥100 members, RR 1.27, CI 1.20‐1.34, 10‐49 vs ≥100 members, RR 0.86, CI 0.80‐0.92, 50‐99 vs ≥ 100 members, *P* < .001), PQRS participation (RR 1.12, CI 1.04‐1.19, *P* < .002), and Southern practice location (RR 0.67, CI 0.64‐0.70, Midwest vs South; RR 0.39, CI 0.37‐0.41, Northeast vs South; RR 0.43, CI 0.41‐0.46, West vs South; *P* < .001).

**Table 3:** Negative binomial regression analysis

Provider characteristics	NB IRR (95% CI)	*P*‐value
Male vs Female	1.29 (1.22‐1.35)	**<.001**
Reports quality measures vs does not	1.12 (1.04‐1.19)	**.002**
Region		**<.001**
Midwest vs South	0.67 (0.64‐0.70)	<.001
Northeast vs South	0.39 (0.37‐0.41)	<.001
West vs South	0.43 (0.41‐0.46)	<.001
Years since medical school		**<.001**
1‐10 years vs >25	0.78 (0.73‐0.85)	<.001
11‐24 years >25	1.00 (0.96‐1.04)	.957
Number members		**<.001**
<10 vs >100	1.51 (1.44‐1.59)	<.001
10‐49 vs >100	1.27 (1.20‐1.34)	<.001
50‐99 vs >100	0.86 (0.80‐0.92)	<.001

**Figure 2:**
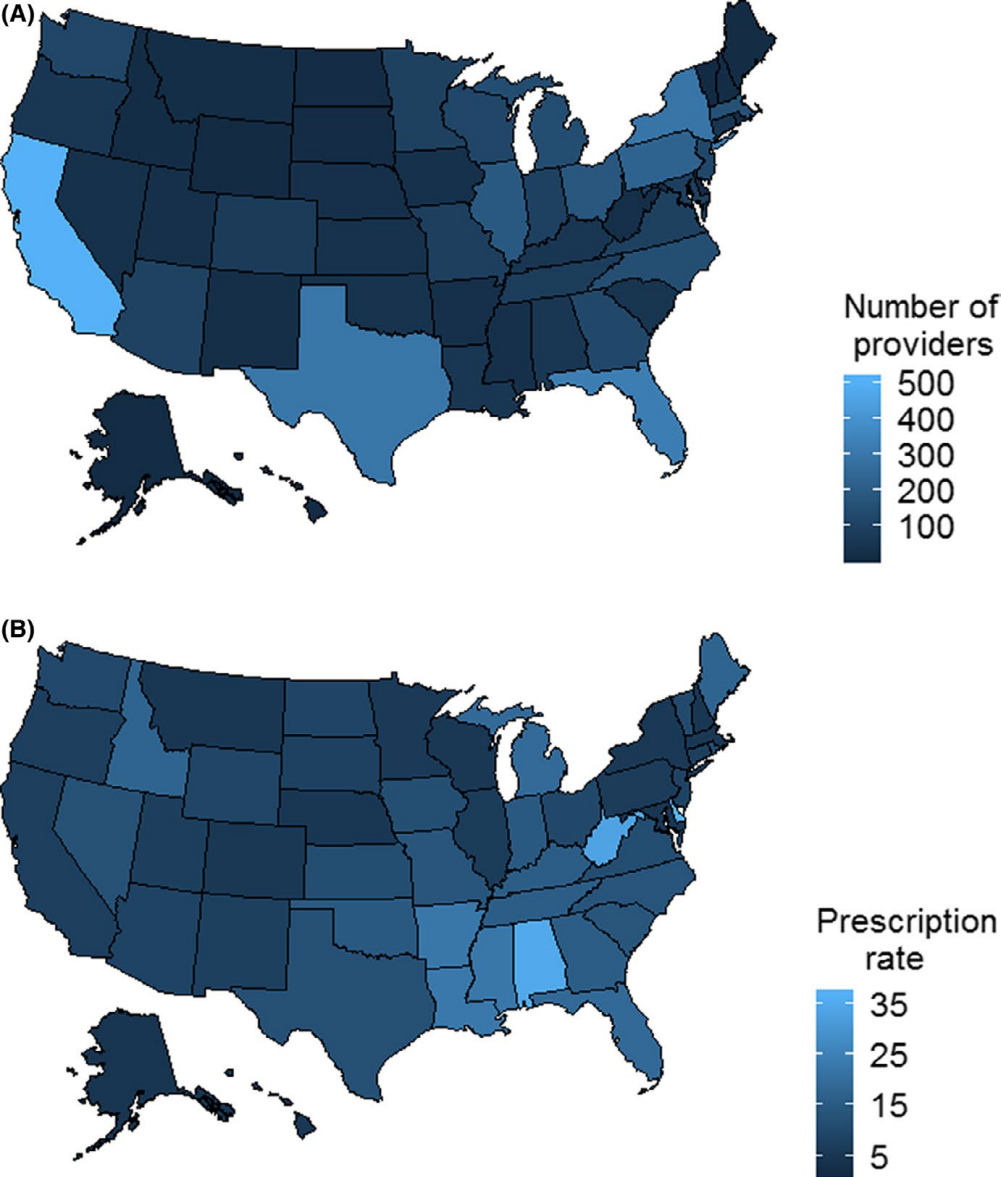
A, Choropleth map of number of practicing ROs per US state in 2016. B, Choropleth map of the average number of RO opioid prescriptions per state in 2016. RO, radiation oncologist

Figure 2A shows the geographic distribution of ROs per state. California had the largest number of practicing ROs. Figure 2B shows the average number of RO opioid prescriptions per state. Delaware had the highest opioid prescription rate (37 opioids prescribed/RO), followed by Alabama (35), West Virginia (33.52), Louisiana (23.07), and Mississippi (22.63) (Table S2). Wisconsin had the lowest opioid prescription rate (5.73 opioids prescribed/RO), followed by Hawaii (5.67), Massachusetts (5.55), Colorado (5.51), and Nebraska (5.43) (Table S2).

## 4 DISCUSSION

In this study of 4627 US ROs practicing in 2016, 61.6% wrote at least 1 opioid prescription and 29.3% wrote at least 10 opioid prescriptions. On multivariable negative binomial and linear regression, male sex, at least 25 years since medical school graduation, group practice size with fewer than 10 members, PQRS participation, and Southern practice location predicted for a greater number of opioid prescriptions.

Among ROs who prescribed an opioid more than 10 times, the average days’ supply prescribed was only 10.5 ± 5.4 days, which suggests that these prescriptions were written for the short‐term management of acute cancer‐related pain for patients undergoing cancer treatment.

The percentage of ROs that prescribed at least 10 opioid prescriptions in 1 year (29.3%) is higher than that seen in some specialties such as ophthalmology (11.2%), dermatology (14.5%), and interventional radiology (12%).^7^, ^8^, ^10^ Given that over half of all patients undergoing cancer treatment and two‐thirds of those with advanced or metastatic disease experience cancer‐related pain, however, this percentage is perhaps disproportionately low.^3^, ^11^, ^12^ Pharmacologic analgesics can effectively alleviate cancer‐related pain in 70%‐90% of cases, yet cancer pain is routinely undertreated, with approximately one‐third of cancer patients receiving insufficient analgesia.^3^

Among patients receiving radiation therapy, cancer pain is also undertreated with over one‐third of these patients reporting inadequately controlled pain. Furthermore, side effects of radiation therapy as well as transportation and immobilization for treatment often exacerbate preexisting, already undertreated cancer‐related pain.^13^ Paradoxically, surveys of radiation oncologists reveal that ROs recognize the prevalence of undertreated pain among their patients, yet do not recommend proportionately strong opioid medications.^5^ Despite acknowledgment of pain undertreatment in radiation oncology and literature emphasizing the importance of pain management education, there has been little improvement in ROs’ comfort with managing pain.^5^

Since the majority of patients receiving radiation therapy are concurrently followed by a medical oncologist, it could be suggested that ROs are deferring opioid prescribing to their medical oncology colleagues. Yet medical oncologists write disproportionately low numbers of opioid prescriptions relative to other specialties. From 2006 to 2014, general practice, family practice, and internal medicine physicians made up 27.4% of US physicians but wrote 35.1% of all opioid prescriptions.^14^ By contrast, hematology/oncology physicians made up 2.5% of all US physicians but only wrote 1.3% of all opioid prescriptions.^14^ It could also be suggested that ROs are limiting opioid prescriptions due to fears about opioid misuse or addiction. While cancer patients are not free of opioid use disorders, studies suggest that cancer patients may be at less risk for opioid dependency and abuse than those in the general population. Opioid‐related death is 10 times less likely to occur in cancer patients than in the general population.^15^ Similarly, opioid‐related hospitalizations among cancer patients are rare and have increased at a low rate over time, in contrast to the spike in opioid‐related hospitalizations in the general population.^15^, ^16^

Our study demonstrated that male ROs prescribed significantly more opioids than their female counterparts. This trend was also found in opioid prescriptions of dermatologists, otolaryngologists, and emergency medicine physicians, though opioid prescriptions did not vary significantly based on physician sex among interventional radiologists.^7^, ^8^, ^17^, ^18^, ^19^ Multiple factors likely underlie our observation that females write fewer opioid prescriptions than males. Female ROs may subspecialize in areas that may require fewer opioid prescriptions or in populations that are not captured in this dataset (ie, pediatrics).^20^, ^21^ Female ROs may also be more likely to refer to pain or palliative care specialists. Previous studies have shown that women in other specialties are more likely to adhere to clinical guidelines, engage in shared decision‐making, and practice value‐based care than men.^22^, ^23^, ^24^ Since pain management is a complex, highly regulated, and patient‐centered endeavor, these characteristics may explain some of the discrepancies in prescription patterns. Male ROs have been shown to submit more charges to Medicare as compared to females; thus, an alternative possibility is that female practitioners see fewer patients compared to their male collegues.^25^

ROs who graduated from medical school at least 25 years ago prescribed significantly more opioids than those who graduated 1‐10 years ago. The same trend was observed in interventional radiology, where physicians who were in practice for greater than 10 years prescribed more opioids on average.^8^ However, years in practice did not significantly change the average number of opioid prescriptions per physician in dermatology.^18^ Medical school training influences physician comfort with pain management and recent changes in attitudes toward opioids may have played a role in reducing the average number of opioid prescriptions for physicians more recently out of school.^5^

ROs at group practices with fewer than 10 members prescribed the highest average number of opioid prescriptions (15.5 ± 44.6), followed by ROs at group practice sizes of 11‐49 members (13.3 ± 25.9), 50‐99 members (8.5 ± 12.7), and ≥100 members (8.8 ± 26.9). While this observational study cannot establish causation, potential reasons for this decrease may be the availability of palliative care or pain management specialists at larger practices. Furthermore, the growth of larger practicing groups has been associated with relatively greater growth in the number of multispecialty practice groups. Physicians independent of specialty are increasingly practicing in larger, multispecialty groups and are more likely to be young and female.^26^ In larger practice environments with pain management specialists available, ROs may refer patients instead of prescribing opioids themselves. This may also be due to previously studied setting‐based prescribing trends. University settings and minority‐specific clinics have higher rates of undertreatment than community‐based treatment settings.^27^ The lower opioid prescribing rates at larger practices found in our study may be reflective of university setting‐associated pain undertreatment. Our study also found that ROs at least 25 years out of medical school wrote the highest average number of opioids (13.3 ± 40.9) compared to ROs 11‐24 years (12.6 ± 26) and 1‐10 years (4.5 ± 11.5) out of medical school. Younger practitioners tend to join large medical groups (defined as larger than 50 members), while smaller medical groups are increasingly composed of older practitioners.^28^ These trends are true for both general and specialty practitioners.^26^ Bringing these points together, our finding that ROs at the smallest medical groups prescribe the highest average number of opioids may be related to smaller practices having the highest proportion of older practitioners.

PQRS participation was also associated with higher numbers of opioid prescriptions. In 2016, quality measures were still reported using the PQRS system, which has since transitioned to the Merit‐based Incentive Payment System under the Quality Payment Program. Under PQRS, measures specific to different specialties were reported to CMS, linking reimbursement to quality of care provided. Efforts to improve these measures could have influenced care decisions, as previous studies have shown CMS compensation changes can drive changes in clinical decision making.^29^, ^30^, ^31^ PQRS measures pertinent to radiation oncology include evaluating pain intensity, plan of care for pain, and whether pain was brought under control within 48 hours. Considering the prevalence of undertreated cancer pain, it is interesting that CMS policy changes emphasizing pain management for radiation oncology patients increased opioid prescriptions in PQRS‐participating practices.

Finally, our study found that ROs practicing in the South prescribed opioids at a higher rate as compared to those in other regions of the US This may be attributed to the low number of ROs in proportion to the overall population in the South. Yet, though the ratio of ROs to overall population is small, southern states have a proportionate number of pain specialists, perhaps indicating a difference in how cancer‐specific pain is treated.^32^, ^33^, ^34^ In our study, states with the highest rates of opioid prescriptions also had low RO‐to‐state population ratios; however, notable outliers exist. For instance, Hawaii has 15 providers for a population of 1.4 million, but was the second lowest in terms of opioids prescribed per RO. By contrast, West Virginia has 27 providers for a population of 1.8 million, but is second highest in opioids prescribed per RO. High opioid prescriptions per RO in the South are consistent with previous studies analyzing opioid prescribing practices among physicians.^8^, ^10^ Such variance has been previously attributed to differences in local medical subcultures reinforced by policies of licensing boards and state‐wide regulations, in addition to the prevalence of managed care.^8^ Further study is needed to understand the impact of rural vs urban practice location on prescription patterns.

### 4.1 Limitations

Our study should be interpreted in the context of the databases used. The Medicare Physician Compare National Downloadable File used in this study contains information only on Medicare‐accepting physicians practicing in the US in 2016. Likewise, the Medicare Part D Prescriber PUF contains information only on patients covered under the Part D drug plan. While the Medicare patient population represents a significant proportion of the US patient population, these findings may not be generalizable to those without Medicare insurance. Additionally, since all provider and patient information associated with fewer than 10 prescriptions were suppressed, our calculations may be slightly affected. As is the case with any single‐payer database, limitations in the Physician and Other Supplier PUF preclude adjusting for potential confounders that are not included in the databases, including disease sites treated, academic affiliation, and clinical volume, which may have been associated with the covariates identified. Since observational data in this study were limited to 2016, as it was the most recent available data at time of analysis, additional studies comparing these data across multiple years would be useful to understand larger trends in opioid prescribing behaviors, especially considering recent changes in opioid prescription regulations and the stigma of opioid use. Lastly, since information about specific patient cases was removed from this database, we were not able to assess appropriateness of opioid prescriptions or to evaluate any patient characteristics associated with ROs’ prescribing patterns.

## 5 CONCLUSIONS

ROs are responsible for managing pain in their patients yet little is known about their opioid prescribing behaviors. Understanding these behaviors provides the foundation for identifying specific recommendations for reducing opioid overprescribing while maintaining pain management options for cancer patients. Our study revealed that sex, practice size, location of practice, and years since medical school graduation were associated with differences in opioid prescriptions. The underlying reasons for these associations are not clear and further investigation is needed to establish opioid prescribing best practices within radiation oncology.

## CONFLICT OF INTEREST

The authors report no conflict of interest.

## AUTHOR CONTRIBUTIONS

Dr Raldow had full access to all of the data in the study and takes responsibility for the integrity of the data and the accuracy of the data analysis, and was involved in concept and design and administrative, technical, or material support. All authors were involved in acquisition, analysis, or interpretation of data, and in critical revision of the manuscript for important intellectual content. Huang and Raldow were involved in drafting of the manuscript. Grogan was involved in statistical analysis. Martin and Raldow were involved in supervision.
