# Supplementary material for: Opioid prescription patterns among radiation oncologists in the United States
Source: Cancer Med. 2020 Mar 13;9(10):3297–304. doi: 10.1002/cam4.2907 (PMC7221425; doi:10.1002/cam4.2907)
Supplement: Supplementary file 1 [file CAM4-9-3297-s001.docx]

**Supplementary Table 1.** Multivariable linear regression of opioid prescriptions per radiation oncologist in 2016

| **Provider characteristics** | **Effect (95% CI)** | **p-value** |
| --- | --- | --- |
| Male vs Female | 4.4 (2.3, 6.4) | **<0.001** |
| Reports Quality Measures vs does not | 3.6 (1.0, 6.2) | **0.006** |
| Region |  | **<0.001** |
| Midwest vs South | -4.7 (-7.2, -2.3) | <0.001 |
| Northeast vs South | -8.4 (-10.9, -5.9) | <0.001 |
| West vs South | -7.6 (-10.1, -5.2) | <0.001 |
| Years since medical school |  | **<0.001** |
| 1-10 years vs > 25 | -5.8 (-8.4, -3.1) | <0.001 |
| 11-24 years > 25 | 0.2 (-1.8, 2.2) | 0.846 |
| Number members |  | **<0.001** |
| <10 vs > 100 | 6.4 (4.1, 8.7) | <0.001 |
| 10-49 > 100 | 3.4 (0.9, 6.0) | 0.008 |
| 50-99 > 100 | -0.02 (-3.4, 3.3) | 0.995 |
|  |  |  |
|  | **p-values for main effects are bolded** | |
