# Supplementary material for: Opioid prescription patterns among radiation oncologists in the United States
Source: Cancer Med. 2020 Mar 13;9(10):3297–304. doi: 10.1002/cam4.2907 (PMC7221425; doi:10.1002/cam4.2907)
Supplement: Supplementary file 2 [file CAM4-9-3297-s002.docx]

**Supplementary Table 2.** States with highest and lowest average opioid prescription rates (opioid prescriptions per RO) per state in 2016.

| **State** | **Population in 2015** | **Average rate (Opioids prescribed/RO)** | **Number of ROs** |
| --- | --- | --- | --- |
| States with highest average opioid prescription rates (opioid prescriptions per RO) per state in 2016 | | | |
| Delaware | 945,934 | 37 | 14 |
| Alabama | 4,858,979 | 35 | 58 |
| West Virginia | 1,844,128 | 33.52 | 27 |
| Louisiana | 4,670,724 | 23.07 | 57 |
| Mississippi | 2,992,333 | 22.63 | 30 |
| Arkansas | 2,978,204 | 22.6 | 30 |
| Michigan | 9,922,576 | 19.4 | 146 |
| Florida | 20,271,272 | 19.2 | 329 |
| Idaho | 1,654,930 | 17.63 | 19 |
| Maine | 1,329,328 | 17.63 | 16 |
| States with lowest average opioid prescription rates (opioid prescriptions per RO) per state in 2016 | | | |
| Wisconsin | 5,771,377 | 5.73 | 120 |
| Hawaii | 1,431,603 | 5.67 | 15 |
| Massachusetts | 6,794,422 | 5.55 | 162 |
| Colorado | 5,456,574 | 5.51 | 77 |
| Nebraska | 1,896,190 | 5.43 | 30 |
| Maryland | 6,006,401 | 5.4 | 92 |
| Montana | 1,032,949 | 5.38 | 21 |
| New Hampshire | 1,330,608 | 5.18 | 17 |
| Alaska | 738,432 | 4.77 | 13 |
| District of Columbia | 672,228 | 1.73 | 26 |
